# Repeated oral exposure to H5N1 influenza virus in pasteurized milk does not cause adverse responses to subsequent influenza infection

**DOI:** 10.1126/sciadv.aeb3906

**Published:** 2025-09-26

**Authors:** Pamela H. Brigleb, Ericka Kirkpatrick Roubidoux, Lauren Lazure, Brandi Livingston, Victoria A. Meliopoulos, Bridgett Sharp, Tyler Ripperger, Shelby Patrick, Dorothea R. Morris, Shaoyuan Tan, Stacey Schultz-Cherry

**Affiliations:** Department of Host Microbe Interactions, St. Jude Children’s Research Hospital, Memphis, TN 38105, USA.

## Abstract

In March 2024, a highly pathogenic avian influenza H5N1 (HPAI) clade 2.3.4.4b virus was identified in US dairy cows, with spillover to cats, poultry, and humans. Up to 30% of commercial pasteurized milk tested contained viral genome copies. The impact of residual viral remnants on host immunity is unknown. Orally ingested proteins can stimulate gut-associated lymphoid tissues, potentially inducing tolerance and altering responses to later infection. We found that milk pasteurization fully inactivated pandemic H1N1 and bovine H5N1 influenza viruses yet preserved hemagglutinin (HA) protein integrity. In mice, repeated oral exposure to inactivated virus did not alter mortality after H5N1 virus challenge. Preliminary data showed that naïve mice exposed to improperly pasteurized milk containing live H5N1 virus developed lethal infection, whereas prior H1N1 infection conferred protection. Mice with preexisting H1N1 immunity remained protected when challenged with bovine H5N1 virus after exposure to H5N1 pasteurized in milk. These findings suggest that pasteurized milk containing inactivated H5N1 virus poses minimal health risks.

## Teaser

H5N1 influenza virus in pasteurized milk does not alter immunity in mice, suggesting limited health risks from consumption.

## INTRODUCTION

Highly pathogenic avian influenza H5N1 (HPAI) viruses have been circulating in wild and domestic poultry populations since the late 1990s including A/Goose/Guangdong/1/1996, resulting in occasional spillover events into mammals, including humans (*1*, *2*). In March 2024, HPAI H5N1 clade 2.3.4.4b was detected in dairy cows and cats in the United States (*3*–*5*). Since the first confirmed infection in March 2024, it has been detected in 17 states as of July 2025 (*6*), with several case reports of spillover into humans, with clinical presentations including conjunctivitis and respiratory illness (*6*–*9*). With the average fatality of ∼50% in previously reported HPAI H5N1 human cases (*10*), it is crucial to understand the drivers of this outbreak in cattle and the potential risks it poses to humans.

One of the initial signs of dairy cattle infectivity was a decrease in feed intake, along with decreased milk production, a condition known as “milk drop” syndrome (*11*, *12*). Upon further investigation, it was found that raw milk can contain high levels of HPAI H5N1 virus and that virus remains infectious on the milking equipment, which may serve as a transmission route between cattle (*13*). This is not the first time influenza has been thought to infect cattle. H1N1 and H3N2 viruses have been previously linked to milk drop syndrome and studies reported seroconversion by hemagglutination inhibition (HAI) assays and virus neutralization assays (*14*, *15*). A recent study reported detection of antibodies to nucleoprotein (NP) and HAI titers to human seasonal H1N1 and H3N2 in cattle tested across 15 states in the United States, which may suggest that influenza genome copies and proteins may be present in pasteurized milk more regularly than previously thought (*16*).

Although raw milk containing infectious virus can pose a threat to exposed workers and other animals or those who consume it, the US Food and Drug Administration maintains that commercial milk that is pasteurized is safe for human consumption. For large batches, milk is typically pasteurized at 72°C for 15 s. Although the collective data demonstrate that there is no infectious HPAI H5N1 virus in commercial milk, several studies have reported the detection of viral genomic material in commercial milk products (*17*). However, there is limited research in understanding how the viral genomic material and remaining influenza viral proteins in pasteurized milk modulate immune responses. Orally ingested viral proteins may be delivered to gut-associated lymphoid tissues (GALTs), where there are three possible outcomes: (i) suppression of systemic immune responses and induction of oral tolerance, resulting in increased disease severity upon infection; (ii) induction of proinflammatory responses boosting and generating protective memory responses; or (iii) little to no impact on response to infection (*18*–*20*).

This study investigates the impact of repeated oral exposure to pasteurized H1N1 virus and a bovine-derived HPAI H5N1 (A/bovine/Ohio/B24OSU-439/2024, or A/bovine/Ohio/24) of clade 2.3.4.4b virus in milk on subsequent influenza infection. This study found that repeated oral exposure to inactivated HPAI H5N1 virus in milk did not cause morbidity or mortality in mice. In contrast, a preliminary observation of improperly pasteurized milk containing live virus led to lethal infection in naïve mice, highlighting the relevance of assessing exposure risks. Mice previously exposed to influenza virus pasteurized in milk did not have enhanced deleterious effects to homologous influenza virus strain. Moreover, mice with prior H1N1 infection immune history that received repeated oral gavage of H5N1 virus pasteurized in milk maintained protection against severe outcomes from H5N1 infections. These studies suggest that repeated oral exposure to influenza virus pasteurized in milk does not induce suppressive immune responses in our model and highlights the safety of ingesting commercial milk positive for H5N1 virus genomic content.

## RESULTS

### Successful heat inactivation of H1N1 and H5N1 viruses

To determine the effectiveness of heat inactivation following the guidelines for milk pasteurization, we conducted several inactivation studies in endpoint polymerase chain reaction (PCR) or real-time PCR machines using the default settings with the lid temperature set to 105°C for A/California/04/2009 H1N1 (CA/09) or an endpoint PCR machine for A/bovine/Ohio/24 H5N1 viruses. Previous studies have found that inactivation of H5N1 virus diluted in milk at 72°C for 15 s is sufficient, whereas others report some inconsistencies among repeats, with the caveat that in-house pasteurization does not fully recapitulate industrial-scale milk pasteurization. To test, PCR-confirmed influenza negative raw milk was obtained from a local dairy source and diluted 1:1 with H1N1 [1.58 × 10^6^ median tissue culture infectious dose (TCID_50_)] or H5N1 (1.58 × 10^8^ TCID_50_) virus and heated at 72°C for 15 to 30 s. Controls included a pasteurized milk-only control to ensure the milk did not lyse the cells or eggs used for titer determination, and a sample where pasteurized milk was mixed with live virus 1:1 before cell or egg infectivity to ensure that the milk did not contain anything that would inhibit viral replication such as anti-influenza antibodies. Samples were inoculated into Madin-Darby canine kidney (MDCK) cells and/or embryonated chicken eggs. Viral inactivation was assessed by TCID_50_ assay at 3 or 5 days postinoculation (dpi) and/or by embryonated chicken eggs assessed for viral positivity by hemagglutination assay for increased viral replication sensitivity.

Using both endpoint and quantitative PCR (qPCR) machines, we found that pasteurization of H1N1 and H5N1 viruses for 72°C for 15 to 30 s reduced viral titers to under the limit of detection (1.5 log_10_/ml) by TCID_50_ in MDCK cells (Tables 1 and 2). After 3 dpi, all inoculated embryonated chicken eggs were negative by hemagglutination assay except for the H1N1 and H5N1 virus only controls (Tables 1 and 2). Pasteurized milk alone did not lyse MDCKs or eggs (Tables 1 and 2), and no significant difference was observed between the virus only controls and live virus added to milk, indicating that the milk itself does not inhibit viral replication (Tables 1 and 2). These results corroborate previous findings (*21*–*26*) and highlight the importance of quality control checks for effective pasteurization for viral inactivation in both cells and eggs. Milk pasteurized in-house at 72°C for 15 s also eliminated bacterial contaminants (fig. S1) to further verify that our pasteurization protocol was sufficient to inactivate both viral and bacterial components in raw milk.

**Table 1.** Characterization of milk pasteurization with H1N1 A/California/04/2009. Experiments were conducted with either an endpoint PCR machine with the lid closed or a real-time PCR machine with the lid closed and set at the default settings of 105°C. Both PCR machines were programmed to a specific input volume to allow the internal temperature of the sample to reach 72°C before the program run. Samples were then either passaged through MDCKs or eggs at 3 dpi. Data are reported as TCID_50_/ml, which was assessed using a hemagglutination assay and calculated with the Reed and Muench calculator, or by the number of eggs that were positive out of the total eggs infected. The limit of detection (LOD) is 10^1.5^ TCID_50_/ml.

		Endpoint PCR machine		qPCR machine	
		Repeat 1	Repeat 2	Repeat 1	Repeat 2
MDCKs	PBS	< LOD	< LOD	–	< LOD
	Pasteurized milk (72°C, 15 s)	< LOD	< LOD	< LOD	< LOD
	CA/09/H1N1	3.16 × 10^7^ TCID_50_/ml	5.62 × 10^7^ TCID_50_/ml	3.16 × 10^7^ TCID_50_/ml	3.16 × 10^7^ TCID_50_/ml
	CA/09/H1N1 added to pasteurized milk	5.62 × 10^7^ TCID_50_/ml	3.16 × 10^7^ TCID_50_/ml	5.62 × 10^7^ TCID_50_/ml	–
Eggs	CA/09/H1N1 pasteurized in milk	< LOD	< LOD	< LOD	< LOD
	Pasteurized milk (72°C, 15 s)	0/3	–	–	–
	CA/09/H1N1	3/3	3/3	–	–
	CA/09/H1N1 pasteurized in milk	0/3	–	0/3	0/3

**Table 2.** Characterization of pasteurization of raw milk and influenza H5N1 A/bovine/Ohio/B24OSU-439/2024. Experiments were conducted with an endpoint PCR machine with the lid closed, programmed to a specific input volume to allow the internal temperature of the sample to reach 72°C before the program run. Samples were then either passaged through MDCKs or eggs. Data are reported as TCID_50_/ml, which was assessed using a hemagglutination assay and calculated with the Reed and Muench calculator, or by the number of eggs that were positive out of the total eggs infected at 36 hours postinoculation for H5N1 control virus or 3 dpi for the rest of the samples. The limit of detection is 10^1.5^ TCID_50_/ml.

		3 days		5 days
		Repeat 1	Repeat 2	Repeat 3
MDCKs	PBS	< LOD	< LOD	< LOD
	Pasteurized milk (72°C, 15 s)	< LOD	< LOD	< LOD
	A/bovine/Ohio/B24OSU-439/2024	3.16 × 10^9^ TCID_50_/ml	3.16 × 10^9^ TCID_50_/ml	3.16 × 10^9^ TCID_50_/ml
	A/bovine/Ohio/B24OSU-439/2024 added to pasteurized milk	3.16 × 10^9^ TCID_50_/ml	3.16 × 10^9^ TCID_50_/ml	–
	A/bovine/Ohio/B24OSU-439/2024 pasteurized in milk	< LOD	< LOD	< LOD
	A/bovine/Ohio/B24OSU-439/2024 pasteurized with milk (72°C, 15 s x2)	–	–	< LOD
	A/bovine/Ohio/B24OSU-439/2024 pasteurized in milk (72°C, 30 s)	–	–	< LOD
Eggs	Pasteurized milk (72°C, 15 s)	0/3	0/3	–
	A/bovine/Ohio/B24OSU-439/2024	3/3	3/3	–
	A/bovine/Ohio/B24OSU-439/2024 pasteurized in milk (72°C, 15 s)	0/3	0/3	–
	A/bovine/Ohio/B24OSU-439/2024 pasteurized in milk (72°C, 15 s x2)	0/3	–	–
	A/bovine/Ohio/B24OSU-439/2024 pasteurized in milk (72°C, 30 s)	0/3	–	–

### Impact of heat-inactivated pasteurization on viral protein stability

Viral genomic material has been detected in commercial milk supplies, with studies indicating that 20 to 30% of tested samples contain H5N1 genomic fragments (*17*, *27*, *28*). Although the presence of these fragments does not pose a disease risk when consumed, the effects of the pasteurization process on viral proteins (as opposed to RNA) remain unclear. Thus, we investigated the stability of viral proteins following pasteurization in milk using our in-house pasteurization protocol and known virus concentrations.

**Fig. 1.**
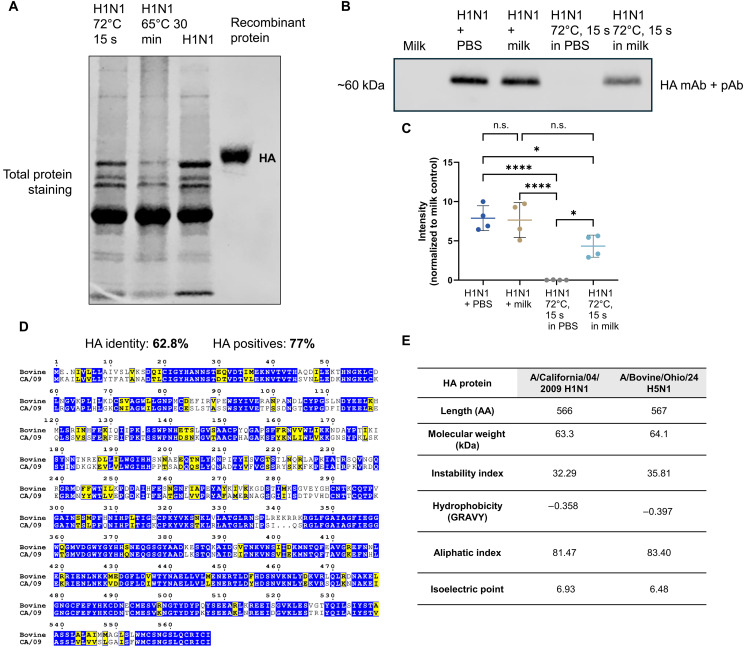
Milk stabilizes influenza virus HA protein during pasteurization. (**A**) Pasteurization of virus at either high temperature (72°C for 15 s) or low temperature (65°C for 30 min). H1N1 CA/09 or recombinant HA protein as a positive control was ran on a 4 to 20% gel and stained for total protein using Coomassie blue. (**B**) Samples were diluted (1:50) of pasteurized milk in PBS, live H1N1 virus in PBS, or H1N1 virus pasteurized in either PBS or milk for 72°C for 15 s and were incubated with a combination of a monoclonal antibody (mAb) and polyclonal antibody (pAb) against the H1N1 HA. (**C**) Analysis of protein expression relative to milk in PBS control. (**D**) HA amino acid sequence comparison between CA/09 H1N1 and A/bovine/Ohio/2024 (H5N1) with identical sequences highlighted in blue and additional similar residues highlighted in yellow. (**E**) In silico analyses of the H1N1 or H5N1 HA amino acid sequences that are important for protein stabilization and melting temperature. Statistical analysis includes (C) one-way ANOVA with Tukey’s multiple comparisons. *P < 0.05; ****P < 0.0001; n.s., not significant. Data are shown as mean with SD.

We first investigated the impact of heat inactivation alone on influenza viral protein stability, using CA/09 H1N1 virus as our model. Viral protein degradation was observed in samples heated by high-temperature pasteurization (72°C for 15 s) that was even more pronounced in low-temperature pasteurization (63°C for 30 min) by total protein stain (Fig. 1A). We next wanted to determine whether heat inactivation in milk would aid in the stabilization of viral proteins, specifically the hemagglutinin (HA) protein, which is the most abundant virus surface protein, and anti-HA antibodies are important in protection from influenza infection (*29*). Milk and milk proteins have been shown to stabilize viruses during heat treatment, likely through mechanisms such as protein coating and pH buffering (*30*–*33*). To test, we used the high-temperature pasteurization in-house protocol with CA/09 H1N1 virus diluted in either phosphate-buffered saline (PBS) or raw milk or added the virus to PBS or now pasteurized milk post–pasteurization treatment. Using HA-specific antibodies, we detected HA in the virus pasteurized in milk, not in PBS, indicating that pasteurization of the virus in milk provides some protection against heat-mediated protein degradation (Fig. 1, B and C).

Recent studies have shown that milk can stabilize HPAI H5N1 virus and alters the thermostability of influenza viruses (*33*). To investigate whether the HA protein stability observed with H1N1 HA viral protein may be also observed with the HA of H5N1 A/bovine/Ohio/24 virus, we used well-established in silico protein models and empirical predictions to assess protein stability under thermal stress. We chose this approach due to BSL3 constraint, limited availability of validated antibodies for heat-treated H5 HA protein, and the likelihood that isolated HA protein would not recapitulate the conformation or protein-milk interactions present in an intact virus particle. The HA amino acid sequence homology between CA/09 H1N1 and the A/bovine/Ohio/24 H5N1 viruses is 62.8% and, with the inclusion of positive residues, 77% (Fig. 1D).

Our analyses included molecular weight and grand average of hydropathy (GRAVY) score that indicates hydrophobicity, aliphatic index, and isoelectric point (*34*, *35*). These parameters can be used to infer thermal stability and structural integrity of proteins. The H1N1 and H5N1 HA proteins have highly similar physical properties across the metrics we assessed (Fig. 1E), including similar hydrophilic profiles, are both stable proteins (instability index < 40), and high aliphatic indices (>80), which are associated with increased resistance to high temperatures and thermostability. The HA proteins also have similar isoelectric points, which suggests the proteins would act similarly in mildly acidic aqueous environments such as in milk. Collectively, these findings support the hypothesis that the H5N1 HA, like H1N1 HA, may retain protein stability following pasteurization in milk, which provide rationale for further investigation and experimental testing. We next investigated whether ingestion of these viral proteins could influence immunity to subsequent influenza infection.

### Live, not inactivated, orally ingested H1N1 virus provides protection against subsequent lethal challenge

**Fig. 2.**
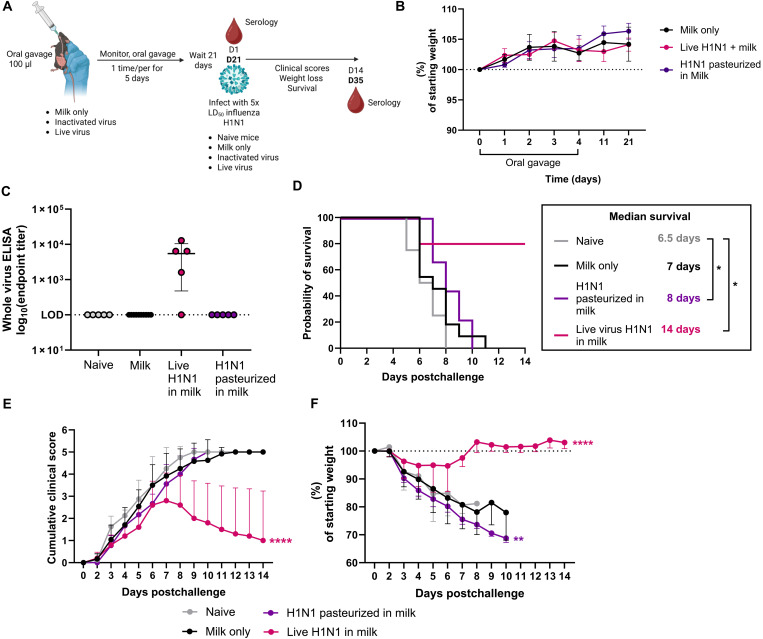
Orally administered live but not inactivated H1N1 virus protects against H1N1 virus challenge. (**A**) Graphical summary of the experimental design. Created in BioRender. Brigleb, P. (2025) https://BioRender.com/btxaiap. (**B**) Weight change during and following oral gavage of pasteurized milk, 10^6^ TCID_50_ live CA/09 H1N1, or virus pasteurized in milk. (**C**) Sera were collected from mice 21 days post–oral gavage start and before challenge. Antibodies against CA/09 H1N1 virus were assessed by ELISA. LOD, limit of detection. (**D** to **F**) Mice were rechallenged with 5× mLD_50_ (5 × 10^3.3^ TCID_50_) 21 days poststart of oral gavage. (D) Survival. (E) Cumulative clinical scores. (F) Weight loss shown as the percent of starting weight. *n* = 5 to 15 mice per group. Statistical analyses include one-way ANOVA with multiple comparisons test (C), log-rank Mantel-Cox test (D), and two-way ANOVA with Dunnett’s multiple comparisons test [(E) and (F)]. *P < 0.05; **P < 0.01; ****P < 0.0001. Data are shown as mean with SD and compared to the naïve control.

Previous studies have demonstrated that oral influenza vaccines may have some effectiveness (*36*–*39*). However, repeated protein exposure to a viral antigen could also lead to development of oral tolerance, thus hindering protective immune responses. We first asked how oral exposure to live virus or virus pasteurized in milk affects response to lethal challenge using the pandemic CA/09 H1N1 virus. Mice were orally inoculated with pasteurized milk only, heat-inactivated H1N1 virus pasteurized in milk, or live virus diluted in pasteurized milk daily for 5 days and monitored for morbidity (Fig. 2A). The virus was diluted at a 1:1 ratio to milk, resulting in the mice receiving a 1.5 × 10^6^ TCID_50_ live or inactivated viral dose daily. All mice gained weight throughout the study and displayed no clinical signs or visible differences in fecal content, such as diarrhea, including the live virus group (Fig. 2B). Twenty-one days post–oral gavage, sera were collected to assess antibody titers and mice were intranasally inoculated with 5× the median lethal dose 50 (mLD_50_) of CA/09 H1N1 virus (5 × 10^3.3^ TCID_50_), including a naïve control group.

We found that oral gavage of live virus, but not virus-pasteurized in milk, induced systemic immunoglobulin G (IgG) antibodies against whole H1N1 virus (Fig. 2C). There were no differences between the milk-only and naïve groups, indicating that milk alone does not provide any protective responses against challenge (Fig. 2D). The presence of systemic IgG antibodies correlated with survival as the mice that had positive IgG titers survived from H1N1 viral challenge. In the live virus group, we observed significant protection from lethal challenge, with improved clinical scores and weight loss compared to the milk-only and naïve groups (Fig. 2, D to F), but no significant impact on mortality or morbidity was observed in the group that received H1N1 pasteurized in milk. Collectively, these results indicate that repeated oral ingestion of H1N1 virus inactivated in milk did not modulate responses to lethal infection, negatively or positively. However, because up to 10^13^ genomic copies/ml have been reported in pasteurized milk, it was important to determine whether these results would be corroborated with bovine-derived H5N1 virus pasteurized in milk.

### Ingestion of inactivated H5N1 in milk does not exacerbate disease after viral challenge

**Fig. 3.**
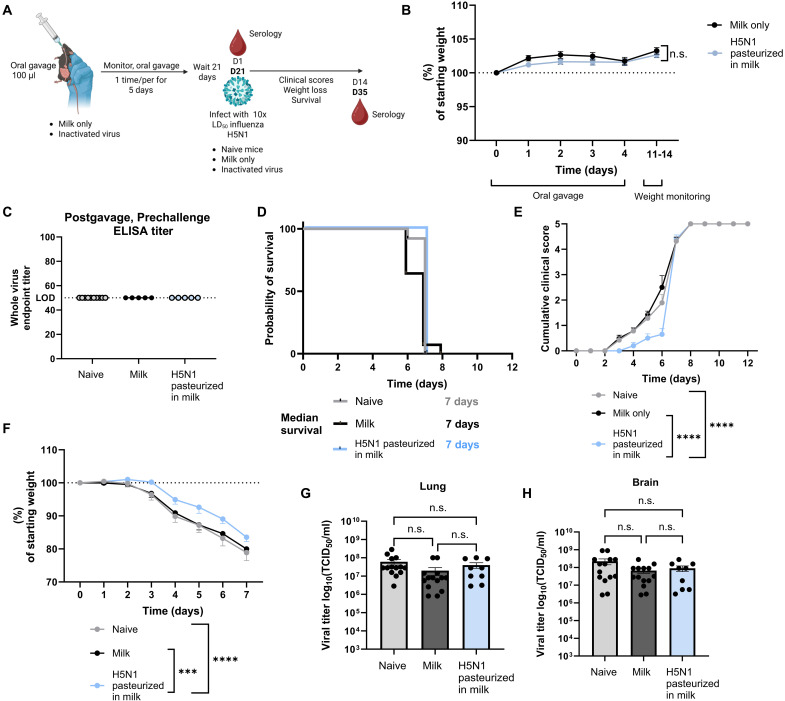
Repeated ingestion of inactivated H5N1 in milk does not worsen disease after viral challenge. (**A**) Graphical summary of the experimental design. Created in BioRender. Brigleb, P. (2025) https://BioRender.com/22e2lg3. (**B**) Weight change during and following oral gavage of pasteurized milk or virus pasteurized in milk. (**C**) Sera were collected from mice 21 days post–oral gavage start and antibodies against whole bovine H5N1 virus were assessed by ELISA. (**D** to **H**) Mice were rechallenged with 10× mLD_50_ 21 days poststart of oral gavage. (D) Survival. (E) Cumulative clinical scores. (F) Weight change. (G) Lung and (H) brain titers at days 6 to 7 postinfection. *n* = 9 to 14 mice per group. Statistical analyses include two-way ANOVA (B), log-rank Mantel-Cox test (C), two-way ANOVA with multiple comparisons [(E) and (F)], and a one-way ANOVA with Tukey’s multiple comparisons test [(G) and (H)]. ***P < 0.001; ****P < 0.0001. Data are shown as mean with SEM.

To test the impact of orally digesting noninfectious H5N1 virus contaminated milk on subsequent exposure to H5N1 virus, mice were orally inoculated with pasteurized milk only or heat-inactivated virus diluted 1:1 in milk daily (1.5 × 10^8^ TCID_50_) for 5 days and monitored for morbidity (Fig. 3). Mice continued to gain weight by 12 days postgavage (Fig. 3B), indicating that repeat ingestion of H5N1 pasteurized in milk did not cause acute disease. At 21 days poststart of oral gavage, sera were collected to determine whether repeated oral exposure of H5N1 pasteurized in milk induced systemic antibody responses. Like the findings with H1N1 virus pasteurized in milk, no systemic antibody titers were detectable to whole virus (IgG) by enzyme-linked immunosorbent assay (ELISA) in the group that received H5N1 pasteurized in milk (Fig. 3C). To determine the effects of repeat exposure to H5N1 pasteurized in milk on subsequent viral infection, mice were inoculated with 10x mLD_50_ (100 TCID_50_) of A/bovine/Ohio/24 H5N1 virus 21 days after the start of oral gavage, with the addition of a naïve control group. The mLD_50_ in male adult mice was 10 TCID_50_ (fig. S2).

All mice succumbed to infection or had to be humanely euthanized by 7 dpi due to neurological presentation (Fig. 3D). Despite the lack of protection against mortality, we observed a delay in morbidity including in cumulative clinical scoring and in weight loss during infection (Fig. 3, E and F). However, we did not observe differences between any groups in lung or brain titers at 7 dpi (Fig. 3H). We further repeated this experiment with a lower lethal challenge (1× mLD_50_) (fig. S3), and the observations were similar to that of the high dose challenge (10× mLD_50_). Collectively, these results suggest that repeated consumption of HPAI H5N1 virus pasteurized in milk, simulating viral genome positive commercial milk, does not adversely affect mortality or morbidity in response to a lethal homologous viral challenge.

### H1N1 immune history confers protection against H5N1, unaffected by oral exposure to inactivated virus

**Fig. 4.**
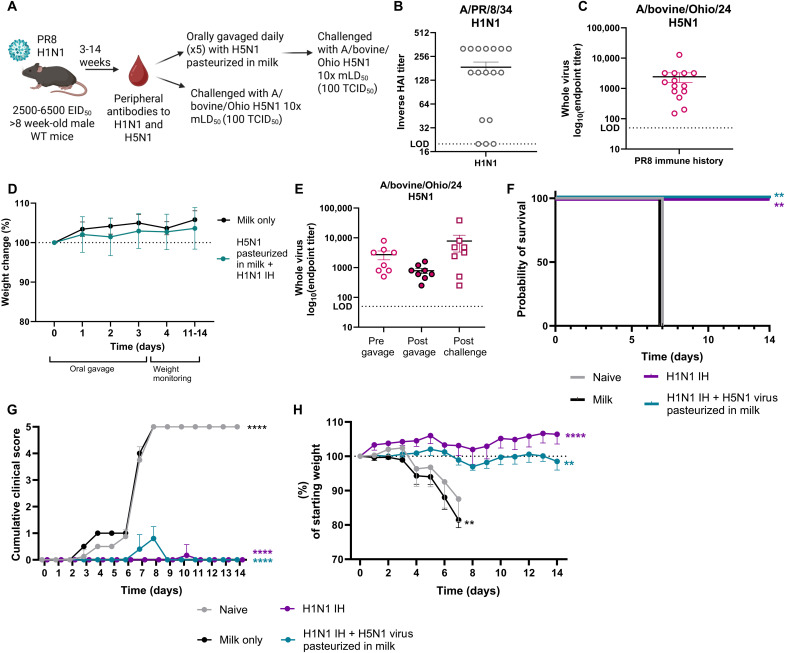
H1N1 immune history protects against H5N1 challenge and is not impaired by ingestion of inactivated virus in milk. (**A**) Graphical summary of the experimental design. Created in BioRender. Brigleb, P. (2025) https://BioRender.com/1h5i5fn. (**B** and **C**) Sera were collected before oral gavage and HAI titers to H1N1 PR8, and whole-virus ELISA titers to bovine H5N1 were assessed, shown as mean with SE. (**D**) Weight change during and following oral gavage of pasteurized milk or virus pasteurized in milk. (**E**) Sera were collected pre- and postgavage and postchallenge, and whole-virus ELISA titers were assessed to bovine H5N1, shown as mean with SE. (**F** to **H**) Mice were rechallenged with 10× mLD_50_ 21 days poststart of oral gavage. (F) Survival. (G) Cumulative clinical scores. (H) Weight change. *n* = 4 to 6 mice per group. Statistical analyses include two-way ANOVA (D), log-rank Mantel-Cox test compared to naïve control (F), and a two-way ANOVA with multiple comparisons [(G) and (H)]. **P < 0.01; ****P < 0.0001. [(D), (G), and (H)] Data are shown as mean with SD and compared to the naïve control.

The studies described above were conducted in naïve mice with no prior exposure to influenza. However, most humans have preexisting immunity to influenza from prior infection or vaccination. To model this, we infected mice with A/Puerto Rico/8/1934 (PR8) H1N1 virus [2500 to 6500 egg infective dose at 50% (EID_50_)], followed 3 to 14 weeks later by either challenge with A/bovine/Ohio/2024 H5N1 or oral gavage with H5N1 virus pasteurized in milk (Fig. 4A). Most mice developed detectable HAI titers against PR8 H1N1 (Fig. 4B), and those that did also exhibited cross-reactive serum antibodies to A/bovine/Ohio/24 H5N1 virus, as measured by whole-virus ELISA (Fig. 4C). For all subsequent analyses, we included only mice with confirmed H1N1 immunity defined as a positive PR8 HAI titer.

Mice with prior H1N1 infection were gavaged daily for 5 days with 1.5 × 10^8^ TCID_50_ of A/bovine/Ohio/24 H5N1 virus pasteurized in milk. Naïve mice receiving milk only served as controls. Neither group exhibited morbidity or mortality following oral gavage (Fig. 4D). In contrast, a batch of virus-containing milk that had undergone insufficient pasteurization and retained low-level infectivity was administered to mice. Although this was not an intentionally repeated experiment, the outcomes were notable: Mice with prior H1N1 immunity exhibited only minor weight loss and survived, whereas naïve mice given the same improperly pasteurized H5N1 virus in milk experienced greater weight loss and 60% mortality (fig. S4), consistent with previous findings (*40*). Although preliminary, these findings suggest that preexisting H1N1 immunity may mitigate disease following oral exposure to low-level infectious H5N1 in contaminated milk. Further controlled studies are needed to validate and mechanistically dissect this observation.

We next investigated (i) whether infection immune history alone from an H1N1 virus provides protection or (ii) whether repeated oral ingestion of H5N1 virus pasteurized in milk would boost or inhibit prior immune history responses against H5N1 viral challenge (10× mLD_50_). Before challenge with H5N1, the serum was collected to determine systemic antibody responses post–oral gavage of H5N1 pasteurized in milk. We did not observe any significant impact on systemic IgG responses to whole virus including those mice that received incomplete pasteurized H5N1 virus in milk (Fig. 4E). Mice with H1N1 immune history only were significantly protected from H5N1 viral challenge, with 100% survival compared to 0% in the naïve group (Fig. 4F). Mice that had H1N1 immune history with repeated oral ingestion of H5N1 virus pasteurized in milk were also 100% protected from challenge of H5N1 (Fig. 4F). Mild clinical scores and weight loss were observed in the group that received repeated oral ingestion of H5N1 virus pasteurized in milk (Fig. 4, G and H) but were still 100% protected from H5N1 virus–induced mortality and had maintained or elevated IgG antibodies 21 days postinfection to confirm infectivity (fig. S4B). Collectively, these results suggest that H1N1 prior immunity provides protection against highly pathogenic bovine H5N1 viral challenge and that ingestion of HPAI contaminated commercial milk does not impede the protective immune responses facilitated by infection H1N1 immune history.

## DISCUSSION

The detection of high levels of HPAI H5N1 virus RNA in commercial milk and other dairy products has raised urgent questions about the potential health risks of consuming virus-contaminated milk. Although pasteurization is presumed to inactivate infectious virus, recent reports suggest that H5N1 can persist in raw or improperly processed milk or on surfaces (*13*, *41*, *42*). Moreover, ingestion of infectious virus, whether experimentally or inadvertently, can lead to systemic infection in animal models, as supported by observations in our study. These findings highlight the potential for oral transmission under certain conditions. However, beyond the risk of infectious virus, the immune consequences of ingesting milk containing noninfectious viral proteins or RNA remain unknown. Understanding how repeated oral exposure to H5N1 viral remnants affects host immunity or disease susceptibility is critical for assessing the safety of milk during ongoing outbreaks of HPAI H5N1 viruses.

Our results demonstrated that the CA/09 H1N1 HA protein retains structural stability following pasteurization in raw milk (Fig. 1), suggesting that viral protein can persist even post–heat inactivation. To evaluate whether the bovine H5N1 HA protein might behave similarly, we used a computational approach to predict its biophysical properties under thermal stress. This approach was implemented due to BSL3 containment restrictions and concerns that isolated recombinant protein would not replicate the HA conformation nor its interactions with milk components compared to a viral particle. Our analysis revealed that H1N1 and H5N1 HA proteins share highly similar properties across multiple predictive parameters, such as hydropathy (GRAVY), aliphatic index, isoelectric point, and instability index (Fig. 1). These properties are known to influence thermal resilience and protein integrity in aqueous environments such as milk. Although these computational predictions must be interpreted cautiously and confirmed experimentally, they suggest that H5N1 HA could remain structurally stable following pasteurization and could therefore persist in dairy products that test negative for infectious virus but remain positive for viral RNA. Moreover, because degradation may limit the effectiveness of antibody-based detection assays, future efforts to monitor viral protein content in milk may benefit from mass spectrometry–based approaches.

Repeated oral exposure to viral proteins can modulate immune responses. This concept is the basis for the development of oral vaccines and several are in the pipeline for influenza (*36*–*39*) and is the basis of oral immunization strategies in livestock (*43*). Ingested viral antigens may elicit protective immunity by generating or boosting antibody or T cell responses against those proteins. Alternatively, it could induce oral tolerance, in which innocuous dietary or microbial antigens are presented by tolerogenic dendritic cells to induce anti-inflammatory, regulatory T cell responses, and systemic hyporesponsiveness (*18*, *44*). If viral proteins are processed in this context, oral tolerance might blunt protective responses or accelerate inflammatory responses upon reexposure to live virus, potentially contributing to worsening outcomes.

To investigate the impact of repeated oral exposure to virus pasteurized in milk, we developed a controlled model in which high titer H1N1 or H5N1 virus was spiked into raw milk and subjected to our in-house pasteurization protocol that follows standard milk pasteurization protocols. This approach allowed us to administer precise and repeatable doses to mice by oral gavage. We selected the highest possible viral titer to administer to evaluate high-exposure conditions. Because of inconsistent results from previous studies with in-house pasteurization protocols, we validated our method using three approaches: a standard 3-day TCID_50_ in MDCKs, an extended TCID_50_ for 5 days, and testing in embryonated eggs. On the basis of these results under these conditions, pasteurization consistently inactivated high-titer H1N1 and H5N1 virus preparations.

The bovine isolate of HPAI H5N1 used in this study is pathogenic in mice with the mLD_50_ of 10^1^ TCID_50_ in adult male mice. Oral administration with a low dose of live virus was sufficient to induce mortality in naïve mice (fig. S4A), consistent with a prior report (*40*). Although our pasteurization protocol was validated and consistently effective, this outcome occurred following a batch of virus that had undergone insufficient pasteurization and had remaining infectious virus. Although unintentional, this provided an opportunity to observe the effects of oral exposure to infectious H5N1 virus. Markedly, mice with a prior PR8 H1N1 viral infection were protected from lethal disease following oral exposure to infectious H5N1 virus–contaminated milk (fig. S4A). These preliminary data indicate that prior influenza immune history may provide protection oral infection of HPAI H5N1 virus. However, further studies are needed to confirm this effect across different types of influenza immune history and viral exposure doses.

There is no evidence that repeated administration of inactivated H5N1 virus in milk induced oral tolerance in mice with or without immune history, with no accelerated disease or mortality compared to naïve mice challenged with HPAI H5N1 virus. PR8 H1N1 immune history alone was sufficient for protection against H5N1 viral challenge. Protection was observed despite only partial sequence homology between PR8 and the bovine H5N1 strain: 65% amino acid similarity in HA and 84% in neuraminidase (NA) (fig. S5). Prior studies have shown that NA-targeting antibodies induced by H1N1 infection can cross-react with H5N1 and contribute to protection (*45*, *46*). Our findings are consistent with this, and we are currently investigating the mechanisms of H1N1 virus mediated cross-protection (*47*). Collectively, these data suggest that prior H1N1 PR8 infection history confers protection against lethal HPAI H5N1 challenge and that this protection is not impeded when repeatedly exposed to noninfectious H5N1 virus containing pasteurized milk.

Although viral RNA has been detected in commercial dairy products (*17*, *24*, *25*), its immunological relevance remains uncertain. Our study presents a tractable model for evaluating the effects of repeated oral exposure to influenza pasteurized in milk and underscores the role of immune history in shaping responses to emerging viral threats. Although further work is needed, including direct testing of H5N1 RNA-positive milk, these results support the safety of consuming pasteurized milk that contains residual viral proteins. More broadly, our findings emphasize the need to better understand how ongoing, low-level exposure to viral antigens through our diet might influence host immunity.

## MATERIALS AND METHODS

### Animal husbandry and ethics statement

All procedures were approved by the Institutional Biosafety Committee and the Animal Care and Use Committee at St. Jude Children’s Research Hospital in compliance with the Guide for the Care and Use of Laboratory Animals (protocol no. 513). These guidelines were developed by the Institute of Laboratory Animal Resources and approved by the Governing Board of the US National Research Council. Mice were kept under 12-hour/12-hour light/dark cycles at an ambient temperature of 20°C and 45% humidity. They had continuous access to diet and water. When required, either for humane endpoints or time point collection, mice were euthanized following the American Veterinary Medical Association guidelines.

### Biosafety

All experiments using the HPAI strain were conducted in a biosafety level 3 enhanced containment laboratory. Investigators were required to wear appropriate respirator equipment (RACAL, Health and Safety Inc., Frederick, MD) and PPE. Mice were housed in negative pressure, High-Efficiency Particulate Air (HEPA)-filtered, and vented isolation containers. Experiments using the pandemic California 2009 H1N1 were conducted in a biosafety level 2.

### Cells and viruses

MDCK cells (American Type Culture Collection, CCL-34) were cultured in Dulbecco’s minimum essential medium (DMEM; Corning) supplemented with 2 mM GlutaMAX (Gibco) and 10% fetal bovine serum (FBS; HyClone) and grown at 37°C with 5% CO_2_. A/California/04/2009 (H1N1) viruses and A/bovine/Ohio/B24OSU-439/2024 were kind gifts from R. Webster and the Webby lab at St. Jude Children’s Research Hospital, respectively. The viruses were propagated in the allantoic cavity of 10-day-old specific pathogen–free embryonated chicken eggs at 37°C. Briefly, allantoic fluid was cleared by centrifugation following harvest and then stored at −80°C. Viral titers were determined by TCID_50_ analysis (see below).

### Pasteurization

Raw cows’ milk used in these studies was tested by qPCR to be negative for both H5N1 and influenza A. Milk with or without virus was pasteurized in PCR tubes with a maximum volume of 50 μl in either an endpoint or real-time PCR machine, as labeled in the figures. Pasteurization occurred for either 72°C for 15 to 30 s or 63°C for 30 min.

### Oral inoculation of mice

Ten- to 16-week-old male C57BL/6J mice (the Jackson Laboratory) were perorally inoculated with sterile disposable plastic oral gavage needles once a day for a total of 5 days. Mice were orally gavage with 100 μl of pasteurized milk diluted in PBS, virus (H1N1 CA/09 or H5N1 A/bovine/Ohio/24) pasteurized in milk, or live virus diluted in pasteurized milk. Mice were monitored and weighed daily at time of gavage and monitored daily after up to 7 days total and then weekly. Fecal contents were also monitored for diarrhea, but no mice displayed signs of intestinal upset or lost weight following oral gavage with pasteurized milk alone.

### Median lethal dose 50 determination

Ten- to 12-week-old male C57BL/6J were lightly anaesthetized with inhaled isoflurane and intranasally inoculated with 10^1^, 10^2^, or 10^3^ TCID_50_ of A/bovine/Ohio/24 H5N1 diluted in PBS for a total volume of 25 μl (two to four mice per dosage). Body weight, clinical scores, and survival were monitored daily for 13 days. Mice were humanly euthanized if they lost more than 30% body weight and/or displayed clinical scores of 3 or above. These included neurological symptoms, which were observed in most euthanized mice, including circling, tremors, and laying on one side with limited motility.

### Viral titer determination

Viral titers were determined as previously described by TCID_50_ assays. Briefly, confluent MDCK cells were infected in duplicate or triplicate with 10-fold dilutions pasteurization samples or tissue homogenates (bead beat in 500 μl of PBS) in 100 μl of minimal essential medium (MEM) plus 0.75% bovine serum albumin (BSA) and tosylsulfonyl phenylalanyl chloromethyl ketone (1 μg/ml)–treated trypsin (H1N1 samples only). After 3 to 5 days of incubation at 37°C and 5% CO_2_, 50 μl of the supernatant was combined and mixed with 50 μl of 0.5% packed turkey red blood cells diluted in PBS for 45 min at room temperature and scored by HA endpoint. Infectious viral titers were calculated using the Reed-Muench method (*48*).

### Mouse viral infectivity

At time points outlined per figure, mice were challenged by intranasal inoculation with either 10× or 1× mLD_50_, as determined in this or previous studies, with A/bovine/Ohio/2024 or 5× the mLD_50_ for A/California/04/2009 diluted in PBS to a total volume of 25 μl that were lightly anesthetized with inhaled isoflurane. Body weight, clinical scores, and survival were monitored daily for 14 days. Moribund mice that lost more than 30% body weight and/or reached clinical scores of greater than 4 were humanely euthanized. Clinical signs were scored as follows: 0, no observable signs; 1, active, squinting, mild hunch, or scruffy appearance; 2, squinting and hunching, ruffled fur, but active; 3, excessive hunching, squinting, visible weight loss, not active when stimulated; 4, not active when stimulated, sunken face and severe weight loss, shivering, rapid breathing, and moribund; and 5, death. For mice infected with H5N1, the majority also displayed neurological signs, including circling, tremors, and laying on one side with limited motility. Mice were humanly euthanized when these neurological signs were present. Posteuthanasia, the whole lung and brain were harvested and stored at −80°C for tissue processing and future analysis.

### Viral growth in embryonated chicken eggs

Ten-day-old embryonated chicken eggs were inoculated with samples described in Tables 1 and 2. All samples were diluted at a 1:2 except for the virus only positive controls and conducted in triplicate. Egg sampling occurred daily with a small aliquot of allantoic fluid and assessed for viral positivity by hemagglutination assay as previously described. At 36 hours postinoculation, the positive control H5N1-infected eggs were killed by incubation overnight at 4°C. The remainder of the eggs inoculated with other samples was allowed to grow for 3 days, at which time the eggs were killed by incubation overnight at 4°C.

### Immunoblots

Samples were processed and loaded on an SDS–polyacrylamide gel electrophoresis (4 to 20% tris-glycine 1.0-mm Mini Protein Gels from Invitrogen). The gel was either stained with Coomassie blue and destained overnight before imaging or was transferred to a nitrocellulose membrane using iBlock transfer stacks (Thermo Fisher Scientific, IB24002). They were then probed for protein with a combination of anti-HA monoclonal and polyclonal antibodies (Sino Biological, 11055-RM10 and 11055-T62). IRDye 680RD goat anti-rabbit IgG secondary (LI-COR, 926-68071) antibody was used with the iBind (Thermo Fisher Scientific) according to the manufacturer’s instructions. The blot was imaged on the LI-COR Odyssey.

### Bacterial screening

Raw milk was pasteurized using the methods described above. In triplicate, raw milk only or raw milk that had been pasteurized were spot plated following serial dilutions on blood agar plates and placed in either aerobic or anaerobic chambers overnight at 37°C. No difference was detected in the colony size or number between the aerobic or anaerobic growth conditions for the raw milk. The number of colonies was enumerated, and the colony-forming units (CFU) per milliliter were calculated. No bacterial colonies were detected under either condition in the in-house pasteurized milk.

### Antibody quantification

Whole-virus (H1N1 or H5N1) ELISAs were conducted using 384-well flat-bottom MaxiSorp plates (Thermo Fisher Scientific) coated with either purified CA/09 at 5 μg/ml or 10^6^ TCID_50_ of the virus stock of A/bovine/Ohio/24 overnight at 4°C. Plates were washed four times with PBS containing 0.1% Tween 20 (PBS-T) using the AquaMax 4000 plate washer system for H1N1 ELISAs or handwashed for H5N1 ELISAs. Plates were blocked with PBS-T containing 0.5% Omniblok nonfat milk powder (AmericanBio) and 3% goat serum (Gibco) for 1 hour at room temperature. The wash buffer was removed, and plates were tapped dry. Mouse sera were diluted 1:5 in PBS and ran in duplicate. Positive and negative mouse sera were used as controls for both sets of ELISAs. Samples were incubated at room temperature for 2 hours and then washed four times with PBS-T. Anti-mouse peroxidase-conjugated IgG secondary antibody was diluted at 1:3000 (Invitrogen, 62-6520) in blocking buffer, and 15 μl was added per well and incubated at room temperature for 1 hour. Plates were washed four times with PBS-T and developed using SIGMAFAST OPD (Sigma-Aldrich) for 10 min at room temperature. Plates were read at 490 nm using a BioTek Synergy2 plate reader and Gen5 (v3.09) software. For each plate, an upper 99% confidence interval (CI) of blank wells OD (optical density) values was determined and used in determining the endpoint titers. Alternatively, mouse sera were treated with receptor-destroying enzyme (RDE; Seiken 370013), and HAI assays were performed as described for H1N1 viruses (*49*, *50*).

### Immune history

Adult male wild-type (WT) C57BL/6J mice were inoculated intranasally with 2500, 4500, or 6500 EID_50_ of A/Puerto Rico/8/1934 (H1N1) and monitored daily for 14 days postinfection. A minimum of 3 weeks postinfection, mice were bled via eye bleeds before oral gavage to determine antibody titers or before challenge with lethal A/bovine/Ohio/24.

### Protein sequence alignment and identity analysis

Amino acid sequences of CA/09 H1N1 (UniProt accession number C3W5S0) and A/bovine/Ohio/24 H5N1 (GenBank: PP836471.1) HA proteins and PR8 H1N1 and A/bovine/Ohio/24 H5N1 HA and NA proteins (UniProt accession numbers P03452 and P03468 and GenBank: PP836471.1 and PP836473.1, respectively) were aligned using Clustal Omega, and the alignment was visualized with ESPript 3.0 (https://espript.ibcp.fr) (*51*). ESPript was used to annotate residue identity and similarity, with conserved residues shaded in blue and similar (positively scoring) substitutions indicated by yellow lettering. This visualization enabled direct comparison of sequence homology across the influenza proteins to assess potential structural and functional conservation.

### Protein prediction and analyses

We used several online tools and databases for protein analyses comparing the A/Ca/09 H1N1 and A/bovine/Ohio/24 H5N1 HA proteins. These include the ExPASy Compute pI/MW tool, ExPASy ProtParam for instability index, estimated isoelectric point, aliphatic index, and GRAVY (*34*, *35*).

### Statistical analyses

All animals were randomly selected for each control and experimental group. Male mice were selected due to current mouse availability, but all experiments should be repeated with female mice to determine whether there are any sex biases in the data. A limitation to this study was the low sample size for some experiments in the supplemental data, due to mouse availability (immune history mice), the urgency of these studies, and limited access to our BSL3 facility. All graphs and statistical analyses were conducted using GraphPad Prism version 10.0 and described in each figure legend if applicable. The sample size is reported per figure. Statistical analyses include a one-way analysis of variance (ANOVA) with Tukey’s multiple comparisons test, two-way ANOVA (mixed model) with multiple comparisons, and a log-rank Mantel-Cox test for survival curves and are also reported in the figure legend.
