# Supplementary material for: Repeated oral exposure to H5N1 influenza virus in pasteurized milk does not cause adverse responses to subsequent influenza infection
Source: Sci Adv. 2025 Sep 26;11(39):eaeb3906. doi: 10.1126/sciadv.aeb3906 (PMC12466813; doi:10.1126/sciadv.aeb3906)
Supplement: Supplementary file 1 — Figs. S1 to S5 [file sciadv.aeb3906_sm.pdf]

Science Advances
AAAS

Supplementary Materials for

Repeated oral exposure to H5N1 influenza virus in pasteurized milk does not cause adverse responses to subsequent influenza infection

Pamela H. Brigleb *et al.*

Corresponding author: Stacey Schultz-Cherry, stacey.schultz-cherry@stjude.org

Sci. Adv. **11**, eaeb3906 (2025)
DOI: 10.1126/sciadv.aeb3906

This PDF file includes:

Figs. S1 to S5

A.

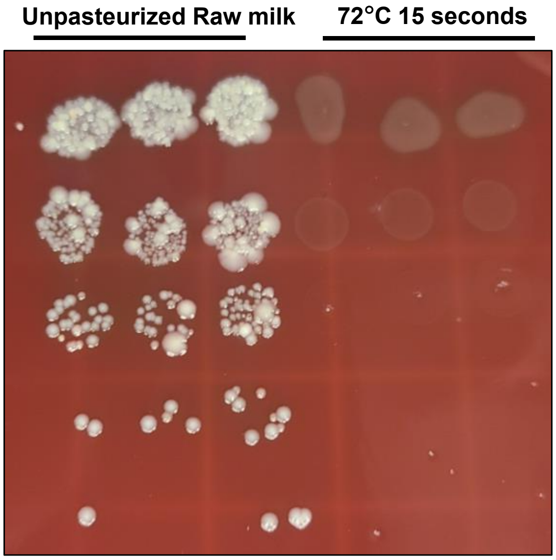

	Unpasteurized milk	72C, 15 seconds pasteurization
**Bacteria concentration**	3.1 x 10^5 CFU/ml	< LOD

Fig. S1. In-house pasteurization protocol successfully kills bacteria in raw milk. Raw milk or milk pasteurized using a real-time PCR machine for 72°C for 15 seconds was serially diluted on a blood agar plate in triplicate and allowed to grow overnight at 37°C.

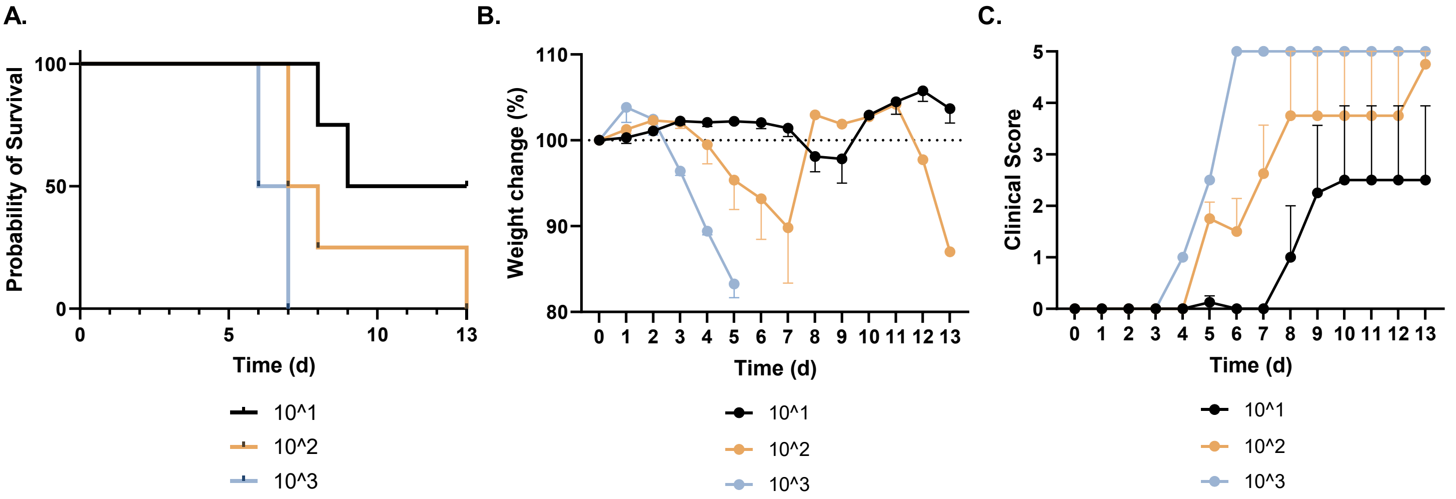

Fig. S2. LD_{50} determination in adult male WT C57Bl/6J male mice. Mice were intranasally inoculated with 10^1 (n=4), 10^2 (n=4) or 10^3 (n=2) $TCID_{50}$ of A/bovine/Ohio.B24OSU-439/2024 H5N1. Mice were weighed and monitored daily, and the majority succumbed to infection displaying neurological symptoms. The LD_{50} in adult male WT C57Bl/6J male mice was determined as 10^1 $TCID_{50}$, and this dose was used for challenge studies with H5N1 marked as LD_{50} challenge. Results represented as mean with standard deviation.

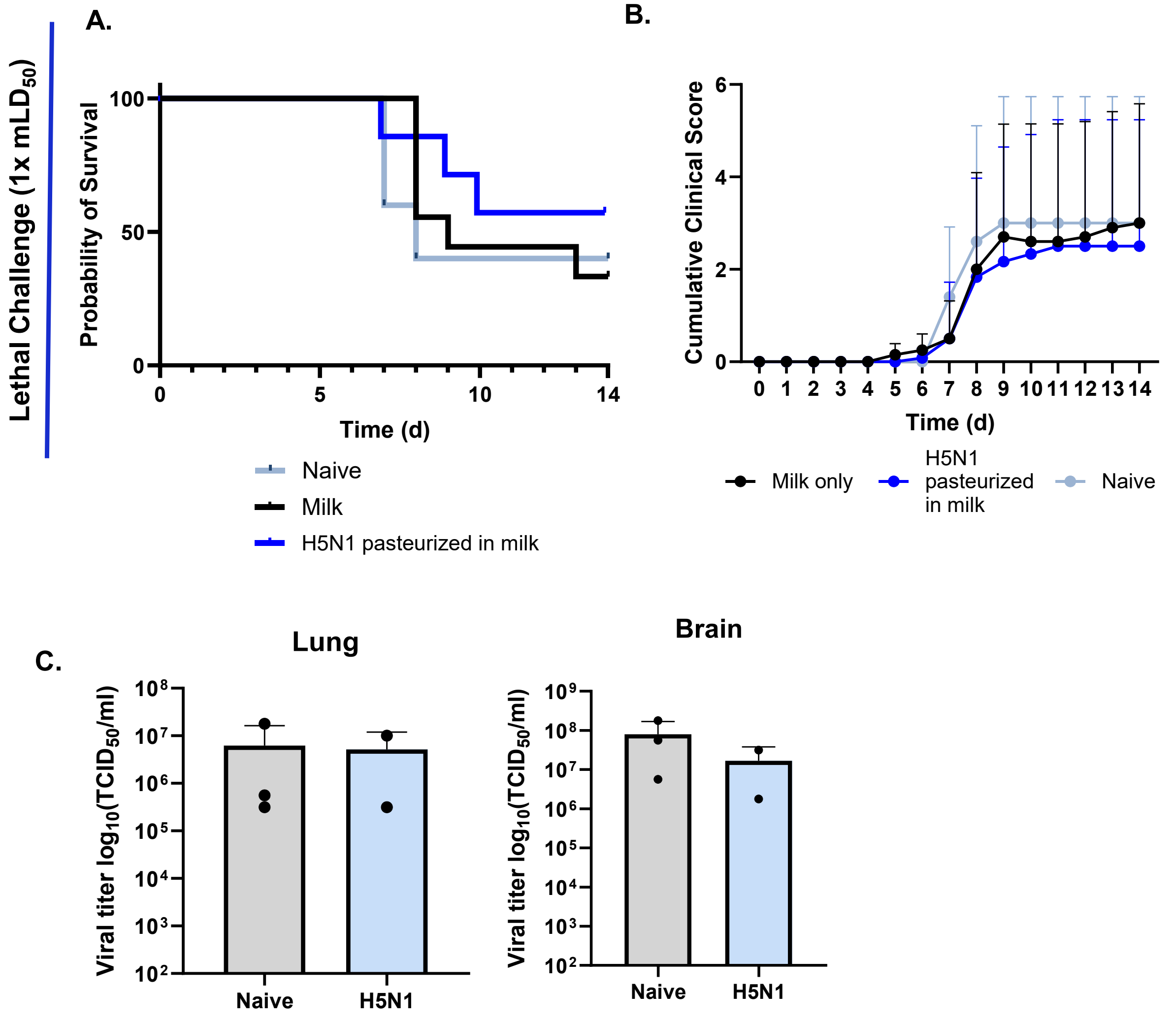

Fig. S3. Repeated ingestion of inactivated H5N1 in milk does not worsen disease after low-dose viral challenge. Mice were orally gavaged with pasteurized milk or H5N1 pasteurized in milk and after 21 days, were rechallenged with 1x mLD_{50} 21 days-post start of oral gavage, including a naïve control group. (A) Survival (B) Cumulative clinical score (C) Lung and brain titers at day 7 post-infection. n=2-9 mice/group, mean with standard deviation shown.

A.

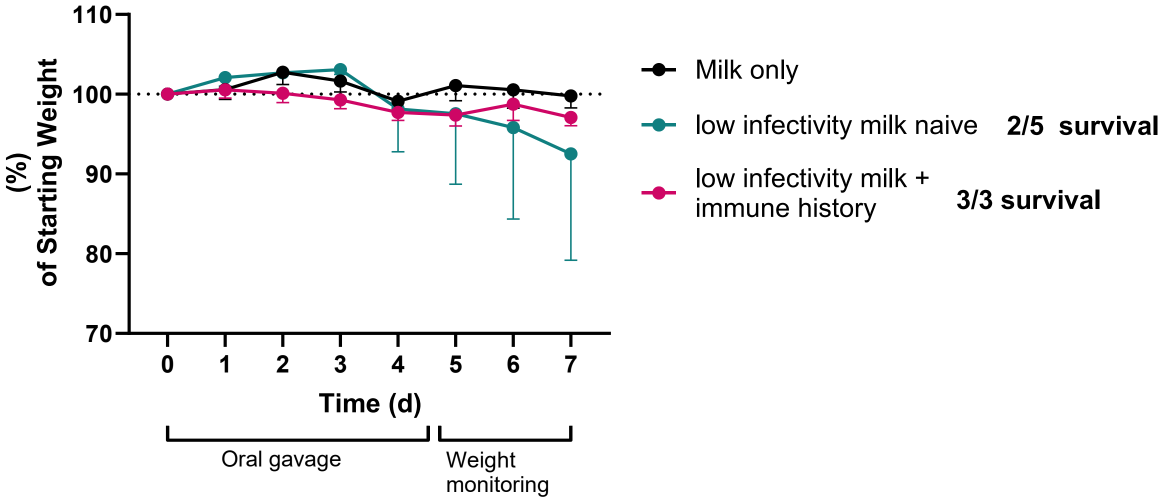

Fig. S4. Improperly inactivated H5N1 in milk causes disease in naïve but not H1N1-immune history mice. Mice were repeatedly gavaged with pasteurized milk or lowly infectious H5N1virus in milk and weight loss and survival was monitored over time. Data shown as mean with standard deviation. n=3/5 mice per group.

A. HA identity: **65%**

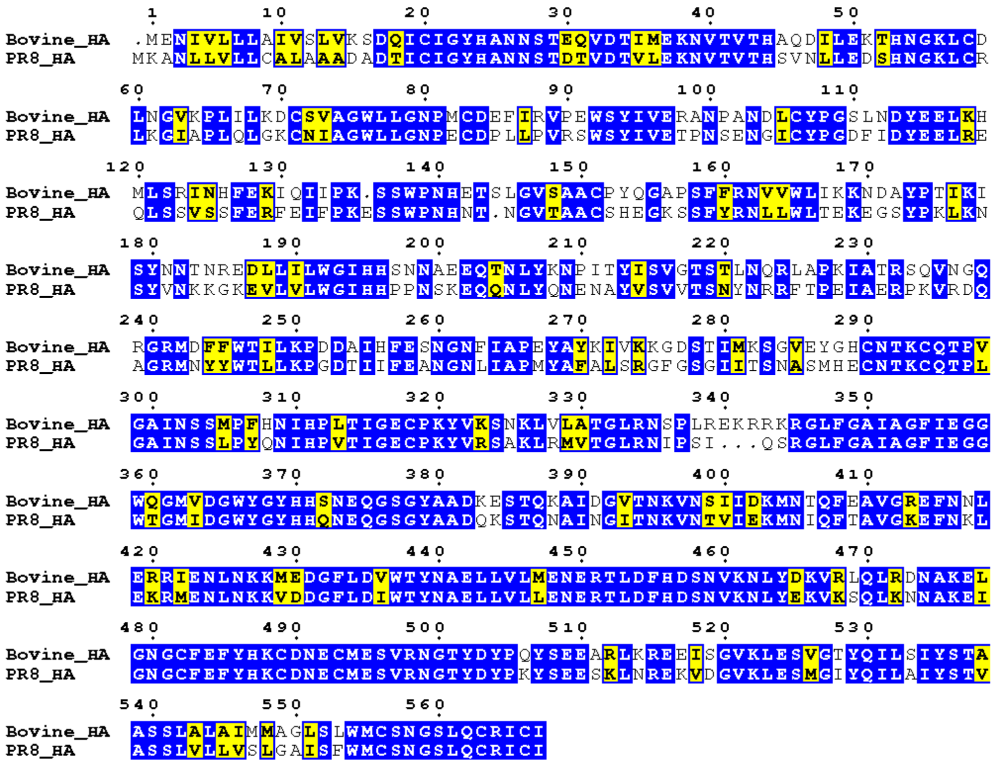

B. NA identity: **84%**

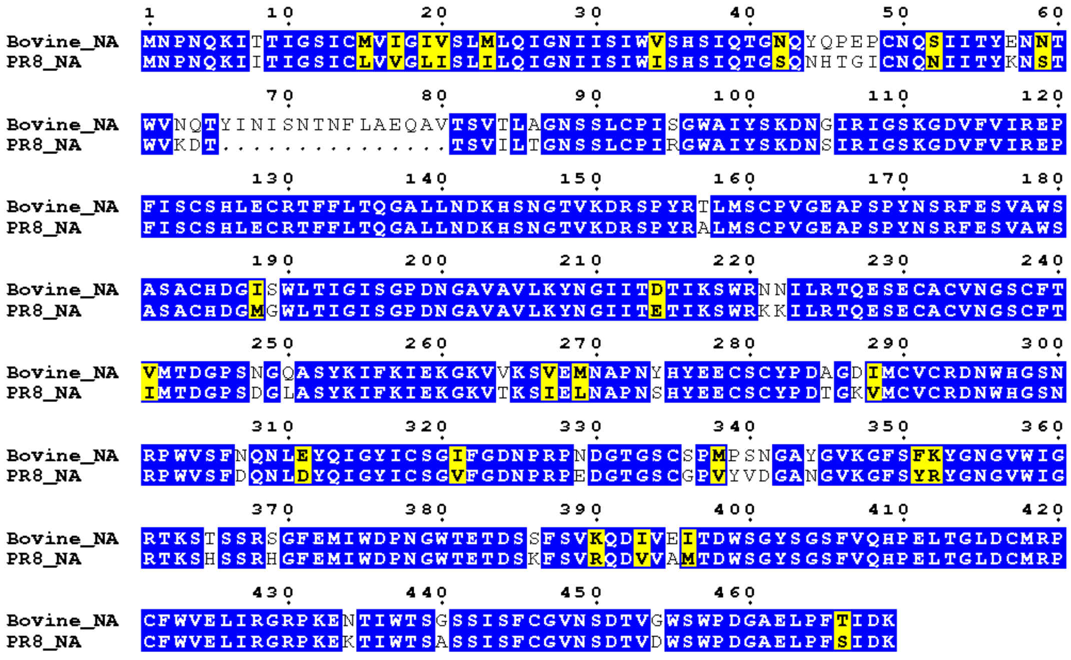

Fig. S5. HA and NA protein sequence alignments. HA and NA protein alignments between A/Puerto Rico/8/1934 H1N1 and A/bovine/Ohio/24 H5N1 with identical sequences highlighted in blue and additional similar residues highlighted in yellow.
